# Sources of variation in maternal allocation in a long‐lived mammal

**DOI:** 10.1111/1365-2656.13243

**Published:** 2020-06-29

**Authors:** Kaitlin R. Macdonald, Jay J. Rotella, Robert A. Garrott, William A. Link

**Affiliations:** ^1^ Ecology Deptartment Montana State University Bozeman MT USA; ^2^ USGS Patuxent Wildlife Research Center Laurel MD USA

**Keywords:** Antarctica, maternal allocation, offspring mass, reproductive effort, Weddell seal

## Abstract

1. Life history theory predicts allocation of energy to reproduction varies with maternal age, but additional maternal features may be important to the allocation of energy to reproduction.
2. We aimed to characterize age‐specific variation in maternal allocation and assess the relationship between maternal allocation and other static and dynamic maternal features.
3. Mass measurements of 531 mothers and pups were used with Bayesian hierarchical models to explain the relationship between diverse maternal attributes and both the proportion of mass allocated by Weddell seal mothers, and the efficiency of mass transfer from mother to pup during lactation as well as the weaning mass of pups.
4. Our results demonstrated that maternal mass was strongly and positively associated with the relative reserves allocated by a mother and a pup's weaning mass but that the efficiency of mass transfer declines with maternal parturition mass. Birthdate was positively associated with proportion mass allocation and pup weaning mass, but mass transfer efficiency was predicted to be highest at the mean birthdate. The relative allocation of maternal reserves declined with maternal age but the efficiency of mass transfer to pups increases, suggestive of selective disappearance of poor‐quality mothers.
5. These findings highlight the importance of considering multiple maternal features when assessing variation in maternal allocation.

## Graphical abstract

Life history predictions were evaluated using a long‐term dataset to characterize age‐specific variation in maternal allocation and the relationship between maternal allocation and other maternal attributes. Results demonstrated evidence for selective disappearance in a long‐lived capital breeder and the importance of including additional maternal attributes when assessing these questions.

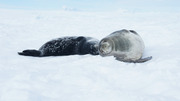

## 1 INTRODUCTION

Maternal allocation, or the energy mothers allocate to reproduction, can have implications for survival and fitness of both mothers and offspring (Lindström, 1999; Mousseau & Fox, 1998). For offspring, reduced growth or limited resources early in life can have consequences for later development, reproduction and survival (Lummaa & Clutton‐Brock, 2002; Ronget et al., 2018; Stauffer, Rotella, & Garrott, 2013), even if compensation for poor early growth occurs (Metcalfe & Monaghan, 2001). Within and among individual variation in maternal allocation can therefore influence population vital rates and is important to our understanding of life history theory and population ecology (Benton, St Clair, & Plaistow, 2008; Clutton‐Brock & Sheldon, 2010). The amount of maternal allocation individual offspring receive can vary due to variation in the environment mothers encounter as well as differences in the attributes of their mothers, which can be either static through life or vary annually. However, few studies simultaneously assess diverse sources of variation in maternal energy allocation to reproduction and how allocation might vary over an individual's lifetime.

Life history theory predicts that reproductive effort should vary with age as the result of a trade‐off among an individual's reproductive output, physiological maintenance and survival given that a finite amount of energy is available for life functions (Stearns, 1992). Several hypotheses that are not mutually exclusive explain how reproductive allocation varies with age. The constraint hypothesis posits that allocation to reproduction is limited by experience, skills and physiological condition such that as individuals age they become more competent in aspects of reproduction (Forslund & Pärt, 1995). Under the restraint hypothesis allocation of resources to reproduction is expected to depend on an individual's residual reproductive value and to vary with age so as to optimize an individual's lifetime fitness (Pianka & Parker, 1975; Williams, 1966). The restraint hypothesis suggests that early in life, when residual reproductive value is high, individuals limit allocation of energy to reproduction to enhance survival and future reproduction (Gadgil & Bossert, 1970). Late in life, when residual reproductive value is low, the terminal investment hypothesis predicts that an individual should increase the proportion of energy that it allocates to reproduction at a cost to future survival or reproduction (Gadgil & Bossert, 1970) and when associated fitness costs are unknown these increases are considered terminal allocation (Weladji et al., 2010). Although evidence for terminal investment has been established in a few experimental studies (Creighton, Heflin, Belk, Moore, & Geber, 2009; Velando, Drummond, & Torres, 2006), more studies of natural populations are needed as previous work on wild animals have either found no support for increases in energetic allocation to reproduction with age (Bowen, Iverson, Mcmillan, & Boness, 2006) or have only been able to identify terminal allocation (Froy, Phillips, Wood, Nussey, & Lewis, 2013; Weladji et al., 2010). In addition to predictions from life history theory regarding age‐specific variation in maternal allocation, it is important to consider possible additional sources of variation that can occur within and among individuals and affect maternal allocation to reproduction.

Heterogeneity in individual quality can result in patterns of reproductive effort across ages that differ at the individual level versus the population level (Cam et al., 2002). Such a difference can occur if individuals within a cohort vary in quality, i.e. latent fitness characteristics, and poor‐quality individuals tend to selectively disappear from the population earlier in life such that the average quality of an individual remaining alive in the population gradually increases as the cohort ages (Forslund & Pärt, 1995; Vaupel, Manton, & Stallard, 1979). If present, selective disappearance can mask underlying age‐specific patterns at the individual level if not considered specifically when evaluating measures of reproductive effort (Beauplet, Barbraud, Dabin, Küssener, & Guinet, 2006; Hamel, Craine, & Towne, 2012).

Age at first reproduction can be an important static predictor of an individual's reproductive success and may affect the relationship between reproductive allocation and maternal age. Long‐lived species that first recruit at an earlier age may suffer costs from reproduction to survival and future reproduction (Gadgil & Bossert, 1970; Reiter & Le Boeuf, 1991). Regardless, more robust individuals might be able to better withstand costs of recruiting to the breeding population at an earlier age (Cam et al., 2002) and exhibit higher reproductive success (Aubry, Koons, Monnat, & Cam, 2009; Bérubé, Festa‐Bianchet, & Jorgenson, 1999).

Other maternal attributes vary through a mother's life and can influence current reproduction or have carry‐over effects on future reproduction. The relationship between maternal attributes and maternal allocation often depends on the reproductive tactic used by the species. There exists a continuum of reproductive tactics used by organisms to meet the energetic demands of reproduction (Jönsson, 1997). On one end of the continuum, income breeders rely on current foraging to meet energetic demands of reproduction. On the other end, capital breeders rely on stores of energy from prior feeding to meet the energetic demands of reproduction (Jönsson, 1997). Because reproduction is costly, an individual's reproductive status in the previous year might influence reproductive expenditures in the current year (Hamel et al., 2012; Pomeroy, Fedak, Rothery, & Anderson, 1999). For capital breeders, individuals skipping reproduction the previous year could accumulate greater body stores and allocate more to the current reproductive attempt (Lunn, Boyd, & Croxall, 1994). The cost of previous reproduction might also vary with individual quality such that low‐quality mothers must skip reproduction to recover body reserves, whereas high‐quality mothers can recover more quickly and sustain consecutive reproductive attempts (Hamel, Côté, Gaillard, & Festa‐Bianchet, 2009). Maternal parturition mass, which can vary among individuals and across an individual's life, is an indicator of the body reserves or resources available to a mother for lactation and maintenance (Gittleman & Thompson, 1988) in species that rely heavily on stored reserves during lactation.

Variables external to a mother can also influence how she allocates her energy to reproduction. In some polygynous species, it is predicted that high‐quality mothers will increase their fitness by allocating more energy during maternal care to male offspring than female offspring (Trivers & Willard, 1973). This sex bias has been observed in several mammal species (Hewison & Gaillard, 1999; Proffitt, Garrott, & Rotella, 2008a). The date of parturition has been found to vary with offspring weight gain during lactation (Plard et al., 2015) as well as with the individual quality of the mother (Rotella, Paterson, & Garrott, 2016). Thus, birth date is likely to be associated with a mother's reproductive allocation. Environmental variation can influence prey availability and abundance, which can, in turn, influence the resources available for reproduction and the body condition of females entering lactation (Crocker, Williams, Costa, & Le Boeuf, 2001) and weaning mass of offspring (Proffitt, Garrott, Rotella, Siniff, & Testa, 2007). In long‐lived species, years of low resource availability are expected to decrease the allocation of resources to reproduction as a means of improving survival rates during challenging conditions (Clutton‐Brock, 1991; Festa‐Bianchet & Jorgenson, 1998).

Studies evaluating variation in maternal allocation must account for diverse factors that can influence an individual's ability to reproduce (Forslund & Pärt, 1995), including static and dynamic attributes of a mother and possible annual variation in environmental conditions. Thus, data on maternal allocation are needed from large numbers of individuals that span a wide variety of ages and that have known reproductive histories. Ideally, measurements of both the energy acquired by mothers as well as the energy allocated to reproduction should be available (Clutton‐Brock, 1984).

Characteristics of the Weddell seal *Leptonychotes weddelli* make this marine mammal a model organism for investigating potential sources of variation in maternal allocation. Variation in reproductive allocation during lactation should reflect the trade‐off between current and future reproduction because lactation is considered the costliest period of reproduction for female mammals (Gittleman & Thompson, 1988). For a capital breeder the ability of an individual to feed and acquire mass each year prior to the reproductive season determines the reserves available to be allocated to offspring, and this is reflected in maternal parturition mass. Weddell seal females are on the capital breeder end of the continuum as they rely primarily on stored body reserves to support the energetic requirements of lactation (Wheatley, Bradshaw, Harcourt, & Hindell, 2008). A mother typically gives birth to a single pup in any given year and is the sole provider of parental care, which permits measurement of energetic allocation that is not complicated by multiple offspring or shared parental care. Therefore, measurement of maternal mass loss during lactation indicates energy allocated to the pup and somatic maintenance (Wheatley, Bradshaw, Davis, Harcourt, & Hindell, 2006). Although some supplemental feeding by females is thought to occur at the end of lactation, the gains for mothers are likely nominal (Wheatley et al., 2008). Pups rely on energy obtained from milk during lactation, and measurement of pup weaning mass is a good indicator of how patterns of maternal allocation are realized by the pup. Due to the strong site fidelity of Weddell seals (Stirling, 1969), accurate reproductive histories are available for a large sample of known‐age females and include information on age at first reproduction, age‐specific parity and reproductive skipping events (Hadley, Rotella, Garrott, & Nichols, 2006).

In addition to the favourable characteristics of Weddell seals, recent studies provide important information regarding several key attributes of maternal allocation in this species. There is evidence of large variation in maternal parturition mass with younger and older mothers weighing less than prime age mothers (Paterson, Rotella, Mannas, & Garrott, 2016). Additionally, younger and older Weddell seal mothers give birth to lighter pups. However, pups born to old females have the highest rates of daily mass gain during lactation (Paterson et al., 2016), which suggests that terminal allocation exists in this species.

In the work presented here, we used mass measurements of mothers and pups directly following parturition, 20 days post‐parturition (mid‐lactation) and 35 days post‐parturition (approximately weaning) to assess predictions of life history theory while also considering other static and dynamic attributes of mothers and accounting for annual variation in maternal allocation due to environmental factors. We assessed multiple aspects of maternal allocation by measuring the proportion of mass allocated by mothers during lactation, and the mass transfer efficiency from mother to pup (pup mass gained per kg of maternal mass lost) during the early lactation period. We note that the mass transfer efficiency metric that we use is likely confounded by individual differences in metabolic rates and activity levels of mothers and pups thus, we measured apparent mass transfer efficiency. We did, however, measure mass transfer efficiency during the early lactation period (~1–20 days post‐parturition) when nearly all mothers were expected to still be nursing their pups and when potential foraging activities were thought to be minimal. In this way we sought to eliminate stronger confounding effects late in lactation due to potential, and very difficult to measure, differences in behaviour and weaning ages. Additionally, we assessed the mass of pups at weaning controlling for the mass of pups at birth, this response variable was chosen because it is a realization of maternal allocation and there is evidence that pup weaning mass is an important determinant of juvenile survival (Proffitt, Garrott, & Rotella, 2008b). The aims of the study were to (a) characterize age‐specific variation in maternal allocation related to life history predictions and (b) assess variation in maternal features that influence maternal allocation.

## 2 MATERIALS AND METHODS

### 2.1 Study system

Erebus Bay is an embayment along the western coast of Ross Island, Antarctica which forms the boundaries of the study area and contains the study population. Sea ice cracks are created and persist from the tidal action where sea ice meets Ross Island and smaller islands within the bay. During the austral spring, female Weddell seals use the sea ice cracks to haul out on the sea ice, forming 8–14 pupping colonies (Stirling, 1969). Mothers typically remain close to pups during the approximately 30‐ to 45‐day lactation period (Tedman & Bryden, 1979). Each year, all pups born in the study population are marked in the interdigital webbing of the rear flippers with individually identifiable livestock tags, and, since 1973, multiple annual population surveys have been done, leading to a population with a large number of adult females whose ages and reproductive histories are known (Cameron & Siniff, 2004).

### 2.2 Sampling design

Data on body mass of mothers and pups throughout lactation were obtained on a sample of animals. Colonies were visited every 24–48 hr throughout each pupping season to identify newborn pups and to associate each mother with her pup. Each year since 2002, attempts were made to weigh a sample of mother–pup pairs at parturition, and later at 20 days after parturition for a mid‐lactation mass and 35 days after parturition for a late‐lactation mass. Not all mother–pup pairs were available for weighing on target dates therefore weights were obtained 1–4 days after parturition, 15–25 days after parturition and 30–40 days after parturition. Pups were weighed using a spring scale or digital weighing platform and mothers were weighed using either a digital weighing platform, photogrammetric methods or both (Ireland, Garrott, Rotella, & Banfield, 2006; Paterson et al., 2016). Due to the difficulty in obtaining maternal mass measurements with a weighing platform throughout lactation, photogrammetry has been used to estimate the mass of Weddell seal mothers since 2002. Photographs of mothers from 2002 to 2010 were taken using a two‐dimensional photogrammetry technique described by Ireland et al. (2006). For photogrammetry data collected during 2012–2016, photographs of mothers were taken and analysed using a three‐dimensional photogrammetry technique described by de Bruyn, Bester, Carlini, and Oosthuizen (2009). For each of the two photogrammetric techniques morphometric measurements were related to mass measurements using linear regression to obtain mass estimates and prediction errors (Appendix S1). Prediction errors were accounted for in modelling of maternal allocation (see Section 2.5).

### 2.3 Response variables

The three response variables associated with the model suites evaluated were proportion mass allocation, mass transfer efficiency and pup weaning mass. The proportion of mass allocated by mothers was calculated by dividing maternal mass loss from parturition to late‐lactation by maternal parturition mass. Mass transfer efficiency was calculated dividing pup daily mass gain by maternal daily mass loss for the early lactation period (~1–20 days post‐parturition). Pup weaning mass was simply pup mass at late‐lactation.

### 2.4 Model covariates

We included combinations of variables thought to vary with the environment and maternal attributes that we predicted would be associated with variation in maternal allocation in three model suites (Appendix S2). Because our primary goal was to evaluate the relationship between maternal energetic allocation and maternal age, maternal age was the key variable of interest in our models. Different patterns of maternal energetic allocation during lactation with age were evaluated using different functional forms of maternal age in candidate models. A model with a linear functional form of age was evaluated to assess whether maternal energetic allocation increased or decreased with age in a consistent fashion. We evaluated two models with nonlinear functional forms of maternal age (quadratic and logarithmic) to assess support for variations of either a minimum at some intermediate age followed by an increase in maternal allocation among old mothers consistent with the terminal allocation hypothesis or a maximum of maternal allocation at some intermediate age followed by a potential decline among old ages. We investigated plots of the raw response variables against maternal age (Appendix S4) for potential breakpoints that would suggest the use of threshold models, but we did not find evidence for their inclusion.

In addition to maternal age, we also included a variety of other covariates in all three of the model suites. Potential correlations among covariates were evaluated and inclusion of all covariates was deemed appropriate. We included maternal parturition mass to control for absolute reserves available to a mother at the start of lactation. We included age of first reproduction as it has been shown to be associated with individual quality of Weddell seals (Hadley et al., 2006). A recent assessment of costs of reproduction in our study population found that there was evidence of reproductive costs to the probability of reproducing in the next year (Chambert, Rotella, Higgs, & Garrott, 2013), which suggests that reproductive effort in the previous year could be important to maternal allocation. Therefore, we included a categorical variable that indicated whether a female was a prebreeder, first‐time breeder, experienced breeder that skipped reproduction the previous year or an experienced breeder that reproduced the previous season to evaluate possible changes in maternal allocation as a function of a female's breeding status in the previous year. At the suggestion of reviewers, birthdate was added to the top model after initial model fitting and selection was completed. Birthdate is related to pup birth mass in this population with the largest pups being born near the peak of pupping (Paterson et al., 2016). Therefore, we included a quadratic functional form for pup birthdate. We included pup sex as a covariate to account for possible differential maternal energetic allocation to male versus female pups. We included pup birth mass in the pup weaning mass model suite to account for the mass of pups at the start of lactation, which is a measure of prenatal maternal allocation. The proportion mass allocation model suite included measurement days as a covariate which accounted for the difference in the timing of mass measurements of mothers. Environmental variation over the course of this study was substantial and included the presence (from 2001 to 2005) of large iceberg fragments in the Ross Sea (Thrush & Cummings, 2011). These fragments had a negative effect on reproductive rates of this population (Chambert, Rotella, & Garrott, 2012), and it is possible that this and other sources of environmental variation led to differences in maternal allocation for females breeding in different years. Accordingly, we included a random effect of year in models to account for environmental variability. Some mothers were sampled in multiple years, and thus, a random effect for mothers was also included in models to account for possible lack of independence among repeated measures of individual mothers.

### 2.5 Statistical procedures

Statistical analysis was done in the R statistical computing environment (R Core Team, 2018), and mass estimation equations for photogrammetry were fit using linear regression (Appendix S1). Maternal mass estimates and associated prediction errors were used in modelling the three response variables. It was important to account for those errors when using mass estimates as covariates in further analyses because mass estimates with unaccounted for measurement error negatively bias estimates of the mass coefficient and diminish the explanatory power of the model (Proffitt, Garrott, Rotella, & Banfield, 2007). Therefore, we used a modelling framework that allowed us to account for measurement error related to photogrammetric mass estimation when evaluating sources of variation in maternal allocation.

We employed a Bayesian modelling framework to assess sources of variation in maternal allocation while accounting for (a) measurement errors associated with maternal masses and (b) the hierarchical nature of our data that included multiple mother–pup pairs per year and repeated measures of some mothers in multiple years. Models were fit in JAGS 4.3.0 (Plummer, 2003) through the R interface using package R2jags (Su & Yajima, 2015). All continuous predictor variables were centred and scaled by 2 standard deviations so that numeric variables could be interpreted on the same scale as binary variables (Gelman, 2008). Model parameters were estimated using Markov Chain Monte Carlo (MCMC). A chain length of 40,000 samples was used for the pup weaning mass models after discarding 20,000 burn‐in samples, a chain length of 70,000 MCMC samples was used after discarding 30,000 burn‐in samples for the adult proportion mass allocation models and a chain length of 250,000 MCMC samples was used after discarding 300,000 burn‐in samples for the mass transfer efficiency models. Chain lengths were increased when running the top model from each model suite with the birthday covariate. Three chains were run in parallel for each model. We assessed model convergence using the potential scale‐reduction factor known as the Gelman–Rubin statistic (Gelman & Rubin, 1992), the Geweke diagnostic, which compares whether the beginning and end of each MCMC are equal and visual inspection of trace plots to confirm model convergence based on functions and outputs from the r package ggmcmc (Fernández‐i‐Marín, 2016). Random effects for year and individual were assumed to be normally distributed around a mean of zero with variance $\sigma_{\text{year}}^{2}$ and $\sigma_{\text{individual}}^{2}$ respectively. We used weakly informative priors for fixed and random effects in the models. For fixed effects we used uniform priors: *U*(−10, 10) for the proportion mass allocation and mass transfer efficiency model suites and *U*(−30, 30) for the pup weaning mass model suite. For the standard deviation of the random effects in both models suites we used uniform priors set to be non‐negative: *U*(0, 50). True maternal masses were included in the models as latent variables. Covariate relationships with mass estimates were evaluated by relating covariates to true maternal mass (Appendix S3).

Some model‐comparison and ‐selection techniques are not appropriate for hierarchical models with complex structures (Hooten & Hobbs, 2015), therefore it was important we used a model‐selection technique that would appropriately handle parameter uncertainty associated with the structure of our models. The Bayesian predictive information criterion (BPIC) is a true Bayesian leave‐one‐out cross‐validation method that evaluates the predictive ability of models and is appropriate for use with hierarchical models (Gelman, Hwang, & Vehtari, 2014; Link & Sauer, 2016). We calculated BPIC values using the procedures set out by Link and Sauer (2016), which involves performing leave‐one‐out cross‐validation (LOOCV) and summing the log conditional predictive ordinates for all observations. The conditional predictive ordinate (CPO) is the probability density of a given observation conditional on the posterior predictive distribution when the observation is omitted (Hooten & Hobbs, 2015). In the proportion mass allocation model structure, our response variable was constructed using multiple maternal mass observations. Thus, for the proportion mass allocation model, BPIC values were calculated by simultaneously omitting each maternal parturition mass and maternal late‐lactation mass. For the pup weaning mass model only the response variable pup weaning mass was omitted. Each cross‐validation analysis was run with a single chain of 10,000 samples that began at a point where convergence had already been achieved, by re‐starting the analysis with the last values obtained from the initial model run. Leave‐one‐out sampling for different observations was done in parallel on multiple cores using the r package snowfall (Knaus, Porzelius, Binder, & Schwarzer, 2009). We performed *z*‐tests on pairs of BPIC values from each model suite to obtain a measure of the magnitude of difference in predictive ability between different models (Link & Sauer, 2016). We assessed goodness‐of‐fit for each of our top models. The squared difference between ordered *z*‐scores was used as a discrepancy measure to assess the mean structure and normality assumption of our models.

We used logistic regression to assess the proportion mass allocation model and the mass transfer efficiency model, therefore coefficient estimates for these models are reported on the log‐odds scale. We also present predicted values for a reference mother with specific characteristics on the real parameter scale to convey results in a biologically meaningful way. A reference mother was an experienced mother that reproduced the previous season and that had mean values for age, age at first reproduction, date of birth and mass at parturition. Reference pup birth mass was set at the mean, and pup sex was set to be female. For reference mothers all random effects were set to zero. When reporting results for coefficient estimates, we present 90% highest posterior density intervals (HDI) from r package HDInterval (Meredith & Kruschke, 2018).

## 3 RESULTS

The final dataset contained 531 observations with information from 362 individual mothers and their pups from 15 different years between 2002 and 2017. Some mothers provided data in multiple years, 249 mothers provided data in only 1 year, 74 in 2 years, 26 in 3 years and 13 in 4 or more years. Masses of pups ranged from 15 to 45 kg at parturition, from 30 to 105 kg at mid‐lactation and from 41 to 142 kg at late‐lactation. Raw mass estimates of maternal masses ranged from 278 to 626 kg at parturition, from 240 to 473 kg at mid‐lactation and from 194 to 402 kg at late‐lactation (Figure S3).

**FIGURE 1:**
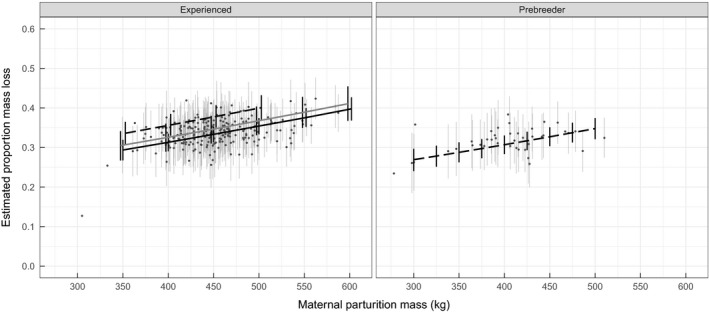
The predicted relationship between proportion of mass lost by a mother during the entire lactation period and maternal mass binned by reproductive status the previous year (experienced and prebreeder), with 90% HDIs of estimates. Solid grey lines indicate an old mother (26 years), solid black lines indicate a prime age mother (17 years) and dashed lines indicate a young mother (8 years). Relationships are shown for combinations of maternal features found in our data. Points represent the true proportion mass allocation estimated from our raw mass estimates with 90% HDIs showing uncertainty around the estimates

Based on our raw mass estimates (i.e. before accounting for error of estimation), the mean proportion of mass allocated by a mother during lactation was 0.33. Raw estimates ranged from 0.07 to 0.48, though the variability of this range is overstated due to measurement error; modelled values were less disparate. The best‐supported model of proportion mass allocation included the quadratic functional form of maternal age (Tables 1 and 2). In the quadratic model, the linear coefficient relating proportion mass allocation to age was negative ( ${\hat{\beta }}_{\text{MaternalAge}}$ = −0.18, *SE* = 0.07) and the quadratic coefficient for age was positive ( ${\hat{\beta }}_{{\text{MaternalAge}}^{2}}$ = 0.17, *SE* = 0.11), which provides some evidence that the proportion of mass allocated during lactation declined slightly with age before increasing at older ages (Figure 1). The top model predicts that an 8‐year‐old mother with the reference covariate values would have a proportion mass allocation of 0.37 (90% HDI: 0.35, 0.40), which would be 165.2 kg (152.8, 177.5) in a typical year. In contrast, a 17‐year‐old mother is predicted to have a proportion mass allocation of 0.33 (90% HDI 0.31, 0.35) and to allocate 145.3 kg (136.6, 154.4). A 26‐year‐old mother is predicted to have a proportion mass allocation of 0.35 (90% HDI: 0.31, 0.38) and to allocate 150.7 kg (135.2, 166.1). Although the model with the quadratic functional form of maternal age was the top model in the suite, the model with a logarithmic functional form of age was the second‐most supported and did not differ in its predictive ability (*p* = 0.67, Table 2). Therefore, predictions from all well‐supported models suggest a decrease in proportion mass allocation of mothers from young to prime ages but differ in the prediction of proportion mass allocation of older mothers (Figure S4). Our inability to distinguish between the functional forms at older ages is likely due to the relative paucity of old mothers in our data (Figure S4) despite our strong efforts to sample all available older mothers in the study population and the additional uncertainty in our estimates that was induced by propagating prediction errors that were associated with our mass‐estimation procedures.

**TABLE 1:** Coefficient estimates (mean of the posterior distribution) for the top model of the three model suites evaluated. Continuous variables were centred using the mean and scaled by two standard deviations. Coefficients for which the 90% highest density interval did not include zero are in bold

Variable	Maternal proportion mass allocated^a^ *n* = 277 Quadratic maternal age model		Pup weaning mass *n* = 478 Logarithmic maternal age model		Mass transfer efficiency^a^ *n* = 235 Quadratic maternal age model	
	Estimate (*SE*)	90% HDI	Estimate (*SE*)	90% HDI	Estimate (*SE*)	90% HDI
Intercept	**−0.672 (0.050)**	**−0.758, −0.586**	**99.14 (1.78)**	**96.23, 102.03**	0.065 (0.128)	−0.148, 0.271
ln(Maternal age)			1.15 (1.84)	−1.88, 4.16		
Maternal age	**−0.184 (0.072)**	**−0.302, −0.065**			**0.380 (0.161)**	**0.114, 0.642**
Maternal age^2^	0.172 (0.109)	−0.008, 0.349			−0.195 (0.274)	−0.639, 0.261
Age primiparity	−0.044 (0.046)	−0.120, 0.031	1.33 (1.40)	−1.01, 3.61	0.038 (0.123)	−0.156, 0.250
Maternal parturition mass	**0.204 (0.047)**	**0.128, 0.283**	**13.99 (1.63)**	**11.26, 16.62**	**−0.363 (0.125)**	**−0.566, −0.155**
pup parturition mass			**7.83 (1.23)**	**5.81, 9.84**		
First‐time breeder last year^b^	−0.059 (0.105)	−0.232, 0.113	1.00 (3.28)	−4.35, 6.43	0.147 (0.354)	−0.384, 0.637
Prebreeder last year^b^	**−0.226 (0.092)**	**−0.375, −0.075**	**−9.40 (2.39)**	**−13.40, −5.53**	−0.142 (0.201)	−0.471, 0.191
Experienced, skipped last year^b^	−0.064 (0.046)	−0.139, 0.012	1.90 (1.32)	−0.30, 4.02	0.053 (0.115)	−0.242, 0.134
Measurement days	**0.123 (0.042)**	**0.055, 0.192**				
Pup sex—male	0.028 (0.041)	−0.039, 0.095	0.06 (1.17)	−1.83, 2.03	−0.007 (0.091)	−0.158, 0.143
Birthdate	**0.112 (0.038)**	**0.049, 0.176**	**2.70 (1.35)**	**0.44, 4.89**	−0.053 (0.103)	**−**0.222, 0.116
Birthdate^2^	−0.007 (0.051)	−0.088, 0.079	−0.89 (1.68)	−3.59, 1.93	**−0.239 (0.153)**	**−0.458, −0.014**

^a^ Reported on the log‐odds scale.

^b^ Reference level is an experienced breeder that reproduced the previous year.

**TABLE 2:** Model‐selection results for the three model suites before including the birthdate covariate. BPIC values are calculated using leave‐one‐out cross‐validation, and higher values reflect greater predictive ability of the model. The top model for each hypothesis model suite is shown in bold. Shown is the *p*‐value from the two‐sided *z*‐test of the difference in predictive ability between the top model (in bold) and the model of that row

Model	Maternal proportion mass allocation suite		Pup weaning mass suite		Mass transfer efficiency suite	
	BPIC value	*z*‐test	BPIC value	*z*‐test	BPIC value	*z*‐test
Null	−2,805.08	0.04	−1,881.41	0.84	−2,381.41	0.02
Linear age	−2,803.85	0.25	−1,882.05	0.06	−2,376.12	0.28
Log age	−2,802.58	0.67	**−1,881.30**	—	−2,374.55	0.62
Quadratic age	**−2,801.95**	—	−1,881.94	0.62	**−2,373.24**	—

Several covariates other than age were associated with proportion mass allocation, and results that follow are based on the best‐supported model. The strongest finding was a positive relationship between proportion mass allocation and maternal parturition mass (Figure 1). Proportion mass allocation for reference mothers weighing 350, 450 and 550 kg are predicted to be 0.302 (90% HDI: 0.277, 0.327), 0.342 (0.323, 0.361) and 0.385 (0.360, 0.409) respectively. Heavier mothers lose a greater proportion of a larger mass, which yields substantial differences in the predicted amounts of mass being allocated to the pup by the 350‐, 450‐ and 550‐kg reference mothers: predicted mass allocation values are 105.6 kg (90% HDI: 97.05, 114.38), 153.9 kg (145.27, 162.45) and 211.7 kg (198.10, 224.85) respectively.

We also found evidence that a female's proportion mass allocation was related to her reproductive status in the previous year (Figure 1). Mothers that were prebreeders the previous year allocated relatively less mass to reproduction compared to experienced breeders. A reference mother that reproduced the previous year is predicted to allocate 0.18 (0.14, 0.22) more than a mother that was a prebreeder the previous year, which translates to a difference of 80.88 kg (61.85, 100.96) of mass allocated when compared to values for prebreeders respectively. Mothers that gave birth later in the season were predicted to allocate a greater proportion of mass: reference mothers that gave birth on 20 October, 27 October and 6 November had predicted proportions of 0.325 (0.291, 0.337), 0.339 (0.320, 0.358) and 0.362 (0.338, 0.387) respectively. There was little evidence that the proportion of mass a mother allocated during lactation was related to her age at primiparity or the sex of her pup (Table 1). Most mothers were only measured in 1 year but repeat observations on 45 mothers allowed us to estimate random effects of individual ( ${\hat{\sigma }}_{\text{Maternal identity}}$ = 0.04). The number of individuals with repeated observations was small therefore the estimated variance associated with individual mothers is imprecise (Figure 4). Sample sizes across 13 years ranged from 3 to 57 individuals allowed us to estimate the variance associated with random effects of year ( ${\hat{\sigma }}_{\text{year}}$ = 0.09), which was modest in size (Figure 4). Overall, our results indicate that young, heavy mothers that are experienced allocate the greatest proportion of mass.

**FIGURE 2:**
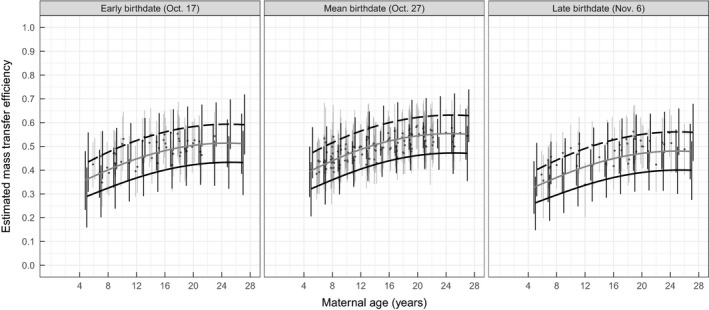
The predicted relationship between mass transfer efficiency and maternal age, binned by early, average and late birthdates with 90% HDIs of estimates. Dashed lines indicate a light mother (350 kg), solid grey lines indicate an average weight mother (450 kg) and solid black lines indicate a heavy mother (550 kg). Relationships are shown for combinations of maternal features found in our data. Points represent the true mass transfer efficiency estimated from our raw mass estimates with 90% HDIs showing uncertainty around the estimates

The mean mass transfer efficiency (kg pup gained/kg mother lost) for the early lactation period (day 1 through 20 of lactation) was 0.496 (range: 0.119–2.624) based on raw mass estimates. The best‐supported model of mass transfer efficiency had a quadratic functional form for maternal age ( ${\hat{\beta }}_{\text{MaternalAge}}$ = 0.380, *SE* = 0.161; ${\hat{\beta }}_{{\text{MaternalAge}}^{2}}$ = −0.195, *SE* = 0.274). Predicted values of mass transfer efficiency from that model increase over most maternal ages but reach an asymptote late in life (Figure 2). Mothers that are 8, 17 and 26 years old with our reference covariate values are predicted to have a mass transfer efficiency of 0.44 (90% HDI: 0.38, 0.51), 0.54 (90% HDI 0.48, 0.59) and 0.56 (90% HDI: 0.47, 0.65) in a typical year.

Maternal parturition mass was negatively related to mass transfer efficiency, and heavier females were estimated to be less efficient at transferring the mass they lost to their pups (Figure 2). Mothers weighing 350, 450 and 550 kg and having reference values for other covariates are predicted to transfer the following proportions of each kg of mass they lose to their pups: 0.59 (90% HDI: 0.52, 0.67), 0.51 (0.46, 0.56) and 0.43 (0.37, 0.49) respectively. Mass transfer efficiency was lower for mothers that gave birth earlier or later than the mean birth date October 27. Estimated values for random effects of year and individuals were both moderate ( ${\hat{\sigma }}_{\text{Maternal identity}}$ = 0.15, ${\hat{\sigma }}_{\text{Year}}$ = 0.15; Figure 4).

The mean weaning mass of pups (35 days post‐parturition) was 99.4 kg (range: 40.6–141.5 kg). The logarithmic maternal age model was the best‐supported model for pup weaning mass. However, the age coefficient was imprecisely estimated ( ${\hat{\beta }}_{\ln\left(\text{MaternalAge}\right)}$ = 1.15, *SE* = 1.84) and had a highest density interval that broadly overlapped zero (Table 1). Further, the quadratic maternal age and null models had similar predictive ability to that of the most‐supported model (Table 2; Figure S6), which limits support for including maternal age in the model.

**FIGURE 3:**
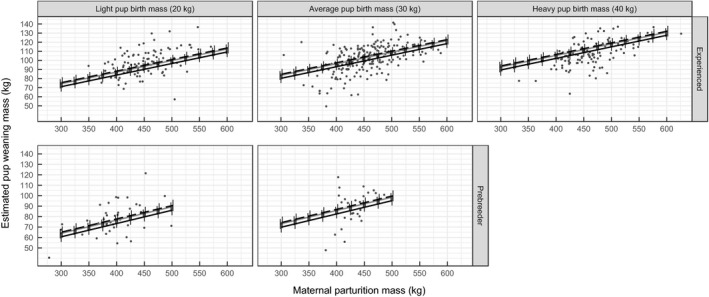
The predicted relationship between pup weaning mass and maternal parturition mass, binned by light, average and heavy pups and maternal reproductive status, with 90% HDIs of estimates. Predictions for experienced mothers are for a 16‐year‐old mother and for prebreeders are for an 8‐year‐old mother. Black solid lines indicate an early birthdate (October 17), grey solid lines indicate an average birthdate (October 27) and dashed lines indicate late birthdates (November 6). Relationships are shown for combinations of maternal features found in our data. Points show the pup weaning masses found in our data

**FIGURE 4:**
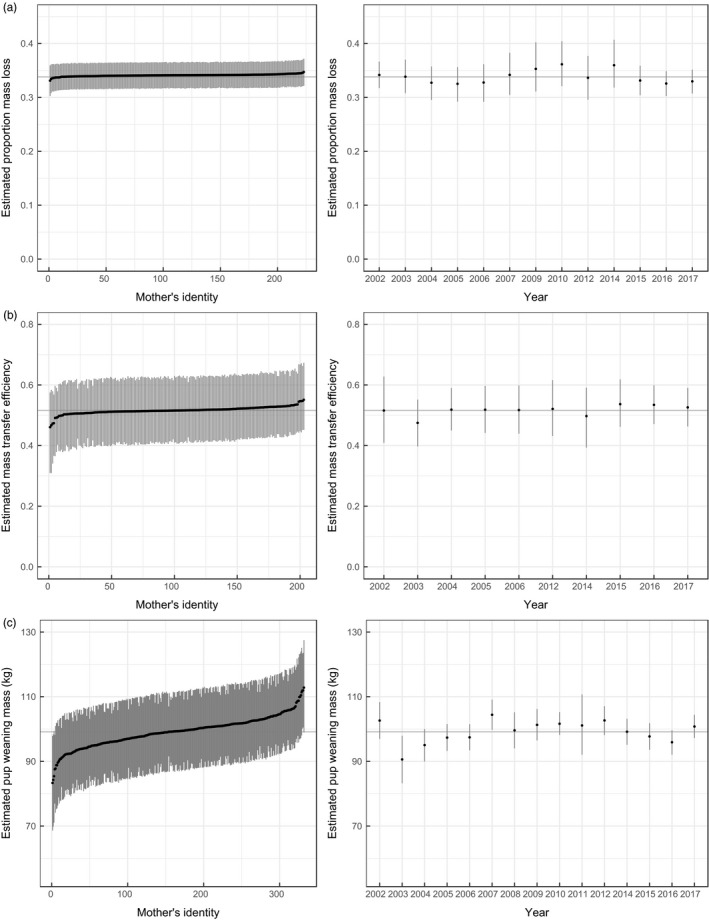
Distributions of estimated random effects with 90% highest density intervals for individual mothers and years from the proportion allocation model (a), the mass transfer efficiency model (b) and the pup weaning mass model (c). The mean is indicated by the grey line

Although there is limited evidence of a relationship between maternal age and pup weaning mass, several covariates were associated with pup weaning mass (Table 1). Maternal parturition mass was positively related to pup weaning mass, and heavier females were estimated to produce pups that were heavier at weaning (Figure 3). Mothers weighing 350, 450 and 550 kg and having reference values for other covariates are predicted to wean pups that weigh: 87.57 kg (90% HDI: 83.79, 91.31), 100.29 kg (97.39, 103.19) and 113.0 kg (109.17, 116.83) respectively. Mothers that were prebreeders in the previous year produced pups that were 9.40 kg (90% HDI: 5.53, 13.40) lighter than those produced by multiparous mothers (Figure 3). Pups born earlier had a lower weaning mass compared to pups born at or after the mean birthdate. The estimated coefficient associated with a pup's mass at parturition was positive (Table 1), such that pups that were heavier at parturition were also heavier at weaning (Figure 3). There was little support for a relationship between pup weaning mass and maternal age at primiparity or pup sex (Table 1). Heavy, older, experienced mothers that gave birth to a heavy pup are predicted to wean the heaviest pups. There was high variance in estimated random effects of pup weaning mass for individual mothers ( ${\hat{\sigma }}_{\text{Maternal identity}}$ = 7.25 kg), with predicted weaning mass ranging from 82.5 kg (90% HDI: 70.0, 95.0) to 113.1 kg (103.2, 123.0; Figure 4). The estimated random effect of year on pup weaning mass was more moderate ( ${\hat{\sigma }}_{\text{Year}}$ = 4.54 kg).

## 4 DISCUSSION

We demonstrated a nonlinear relationship between the proportion of mass allocated by mothers over the course of lactation and maternal age with proportion mass allocation being high among young mothers and declining among prime age mothers. Although the best‐supported model predicts that the proportion of mass allocated to pups increases at the oldest ages, which would be suggestive of terminal allocation, the similar predictive ability of models with other functional forms of maternal age prevent us from making strong inferences regarding the relationship at the oldest ages. As such our results provide only weak support for the terminal allocation hypothesis that posits that old mothers increase allocation to reproduction as their reproductive value declines. The negative relationship between maternal age and proportion mass allocation during early life demonstrated here contradicts the expected increase in maternal allocation with maternal age predicted by the restraint hypotheses, and which has been found in several species (Bowen et al., 2006; Dugdale, Pope, Newman, Macdonald, & Burke, 2011; Ericsson, Wallin, Ball, & Broberg, 2001). The proportion of mass allocated by mothers may not fully reflect maternal allocation if individuals differ with regards to how efficiently they use their body reserves for somatic maintenance and nursing offspring. We found that mass transfer efficiency is positively related to maternal age and this may explain why young mothers allocate a greater proportion of body reserves compared to prime age and old mothers.

Other maternal features were related to the proportion of mass allocated by mothers. We found that maternal parturition mass had a strong, positive relationship with the proportion of mass lost by mothers during lactation. Our finding that heavier mothers lost a greater proportion of mass during lactation is consistent with results from a previous study of Weddell seals, (Wheatley et al., 2006) and a study from another capital breeder (Côté & Festa‐Bianchet, 2001), although others have found a contrasting relationship (Fedak, Arnbom, & Boyd, 1996; Hamel et al., 2012). We agree with suggestions by previous authors (Gittleman & Thompson, 1988) that the increase in proportion of mass allocated by heavier mothers is at least in part because heavier mothers have greater reserves to draw from during lactation and can therefore lose a greater proportion of their reserves possibly before hitting a body composition threshold at which energetic demands of lactation are no longer supported (Carlini et al., 2004). Mothers that were prebreeders the previous year lost the smallest proportion of mass compared to mothers of any other reproductive status. It is likely that primiparous females are not fully developed physiologically (Künkele, 2000) or lack the experience to allocate a commensurate proportion of body reserves to experienced mothers (Broussard, Dobson, & Murie, 2008; Lang, Iverson, & Bowen, 2012).

We found that mass transfer efficiency increased with maternal age before plateauing at the oldest ages such that older mothers were more efficient at transferring mass to pups. We suggest that this increase in mass transfer efficiency is likely due to selective disappearance of poor‐quality mothers from the breeding population. Individual repeatability in milk production and composition has been demonstrated in wild mammals (Lang, Iverson, & Bowen, 2009; Renaud, Blanchet, Cohen, & Pelletier, 2019) and in domestic mammals (Bergsma, Kanis, Verstegen, & Knol, 2008; VandeHaar et al., 2016); and consistent, heritable differences in milk production have been linked to differences in fitness (Gilbert et al., 2012; Spurlock, Dekkers, Fernando, Koltes, & Wolc, 2012). Therefore, if the efficiency of meeting energetic demands of lactation is positively correlated to survival or reproduction, a higher proportion of high‐quality individuals would be found amongst older age mothers. While it is possible that improvements in mass transfer efficiency with age may be due to changes in experience or physiology due to constraint early in life (Forslund & Pärt, 1995), we did not find lower mass transfer efficiency in mothers breeding for the first time, similar to results for grey seals *Halichoerus grypus* (Lang, Iverson, & Bowen, 2011), or for mothers with a later age at primiparity and therefore less reproductive experience, which would be expected if this were the case. Although, we were only able to measure apparent mass transfer efficiency as we were unable to eliminate all confounding effects and it is possible this may have obscured the true patterns of mass transfer efficiency, we largely removed stronger confounding effects due to feeding or differences in lactation lengths. Selective disappearance has been demonstrated in several long‐lived species across multiple aspects of reproduction (Beauplet et al., 2006; Hayward et al., 2013; Zhang, Vedder, Becker, & Bouwhuis, 2015) and suggests there is individual heterogeneity in the quality of mothers.

Besides maternal age, maternal mass was found to be strongly related to mass transfer efficiency. We found a negative relationship between maternal parturition mass and mass transfer efficiency. A similar relationship has been found in the southern elephant seal *Mirounga leonine* (Carlini et al., 2004), and the authors suggest the decreased efficiency of energy transfer with increasing maternal mass was due to a correlation between mass and the proportion of lipid and protein used in milk production versus maternal maintenance. Regardless of the mechanism behind the relationship, this finding suggests that the increased allocation of mass by heavier mothers is offset somewhat by decreased mass transfer efficiency.

Our results did not provide strong support for a relationship between pup weaning mass and maternal age. Our best‐supported model, the logarithmic maternal age model, had similar predictive ability to the null model, which indicates weak support for a logarithmic relationship. A positive relationship between pup mass gain during lactation and maternal age has been demonstrated in our study population (Paterson et al., 2016), but by adding maternal parturition mass to our models we demonstrated that age effects become less important for pup growth during lactation when maternal mass is decoupled from age. Studies of other capital breeders have reported either a lack of relationship between maternal age and offspring weaning mass (Arnbom, Fedak, & Boyd, 1997), or declines in offspring weaning mass related to maternal senescence (Bowen et al., 2006; Hamel et al., 2012). Interestingly, those mothers remaining at older ages weaned pups of similar size to those produced by prime age and younger mothers regardless of their ability to more efficiently transfer mass to offspring, which suggests that mothers may be conservative in allocation of energy to their offspring. Alternatively, there is evidence for large variation among individuals in lactation lengths (Wheatley et al., 2006). Therefore, our use of a standardized 35 post‐parturition weaning date, due to the difficulty in determining weaning, may have underestimated the allocation of some mothers.

We found a positive relationship between maternal parturition mass and pup weaning mass. This positive relationship found by this and other studies (Bowen, Iverson, Boness, & Oftedal, 2001; Hamel et al., 2012; Martin, Festa‐Bianchet, Andrade, & McPeek, 2010; Pomeroy et al., 1999) indicates that maternal reserves are important to the growth of offspring in capital breeders. The mass of pups at weaning and relative maternal allocation is likely tied to both environmental conditions during foraging periods and the ability of mothers to efficiently store resources as body reserves (McMahon, Harcourt, Burton, Daniel, & Hindell, 2017; Proffitt, Garrott, Rotella, Siniff, et al., 2007; Wheatley et al., 2006), therefore this result may be due to heterogeneity among mothers in acquisition and storage of food.

Other attributes of mothers and pups were related to pup weaning mass. Pups from mothers that were prebreeders the previous year were lightest compared to pups from mothers of any other previous reproductive status. Our results add support to findings that primiparous mothers in this population produce pups with lower mass gain during both early and late‐lactation (Paterson et al., 2016). Pup weaning mass increased from early birthdates to the peak of the birth pulse before plateauing at the latest birthdates, which is surprising given recent findings for our study population that suggested that mothers that give birth earliest tend to be of higher quality (Rotella et al., 2016) such that they might be expected to wean larger pups. We found that proportion of mass allocated by mothers increased linearly with birthdate but that mothers that gave birth before and after the peak had lower mass transfer efficiencies. A potential explanation for this finding might be that mothers that give birth later may compensate for a lower mass transfer efficiency by allocating a greater proportion of mass, while mothers giving birth earlier are unable to compensate for a lower mass transfer efficiency. Additional research is needed to determine the mechanism behind this unexpected relationship.

Individual mothers vary little in their relative mass allocation during lactation but vary considerably in the weaning mass their pups reach due to important variation in total mass allocation. It seems that mothers are more constrained in the proportion of mass they lose but exhibit individual differences in mass transfer efficiency and possibly other reproductive attributes that lead to greater variance in the weaning mass of pups. There was moderate variation in the random effect for year for the proportion of mass lost by mothers and pup weaning mass. Weddell seal mothers rely on stored body reserves to meet the energetic demands of lactation therefore it may be that females forego reproduction if they do not have adequate body reserves. Therefore, those mothers that produce a pup have already passed through a filter on breeding probability and likely allocate at least a minimum proportion of reserves to successfully wean a pup, leading to modest variation in allocation at the population level across years.

## 5 CONCLUSIONS

We found evidence for the selective disappearance of poor‐quality individuals from the breeding population, which was exhibited by an increase in mass transfer efficiency with maternal age. There was weak support for the terminal allocation hypothesis that posits that mothers increase reproductive effort as they reach old age. Although we found that the proportion of mass allocated by mothers decreased with maternal age, this pattern could be due to selective disappearance. If so, maternal allocation at the individual level may not change with age, which, if true, could explain our inability to detect a clear relationship between pup weaning mass and maternal age. Our results suggest that the considerable variation in maternal parturition mass is strongly associated with the large differences in the amount of energy allocated to different pups and therefore the weaning masses of pups. Based on previous findings that heterogeneity in individual quality of mothers can lead to large differences in lifetime reproductive output (Desprez, Gimenez, McMahon, Hindell, & Harcourt, 2018), we predict that heavier mothers that are of higher quality, as exhibited by their mass transfer efficiency, will contribute more to the population due to their greater allocation and potentially longer life span. Given previous evidence that weaning mass of pups in Weddell seals is positively related to pup survival during the juvenile period (Proffitt et al., 2008b), and given results for diverse vertebrate species that indicate that mass could affect survival and reproduction later in life (Lummaa & Clutton‐Brock, 2002; Ronget et al., 2018), identifying the relationship between maternal features that influence maternal allocation and offspring survival and reproductive success may be a productive avenue for future research on the determinants of lifetime fitness and associated trade‐offs.

## AUTHORS' CONTRIBUTIONS

J.J.R. and R.A.G. manage the Weddell seal project; K.R.M., J.J.R. and W.A.L. analysed the data; K.R.M. led the writing of the manuscript. All authors contributed critically to the drafts and gave final approval for publication.

## Supporting information

Appendix S1‐S4

## Data Availability

Data available from the Dryad Digital Repository: https://doi.org/10.5061/dryad.f1vhhmgtb (Macdonald, Rotella, Garrott, & Link, 2020).
