# Supplementary material for: Sources of variation in maternal allocation in a long‐lived mammal
Source: J Anim Ecol. 2020 Jun 29;89(8):1927–40. doi: 10.1111/1365-2656.13243 (PMC7497196; doi:10.1111/1365-2656.13243)
Supplement: Supplementary file 1 — Appendix S1‐S4 [file JANE-89-1927-s001.docx]

Appendix S1 - Supporting photogrammetry methods

Photogrammetric mass estimates are based on regression models that relate photogrammetric measurements to mass values from the weighing platform that were obtained for a subset of mothers for which both physical mass and photogrammetric measurements were obtained on the same or a proximal day. Not all physical mass and photogrammetric measurements were obtained on the target day and corrections for the day of measurement were incorporated into our models.


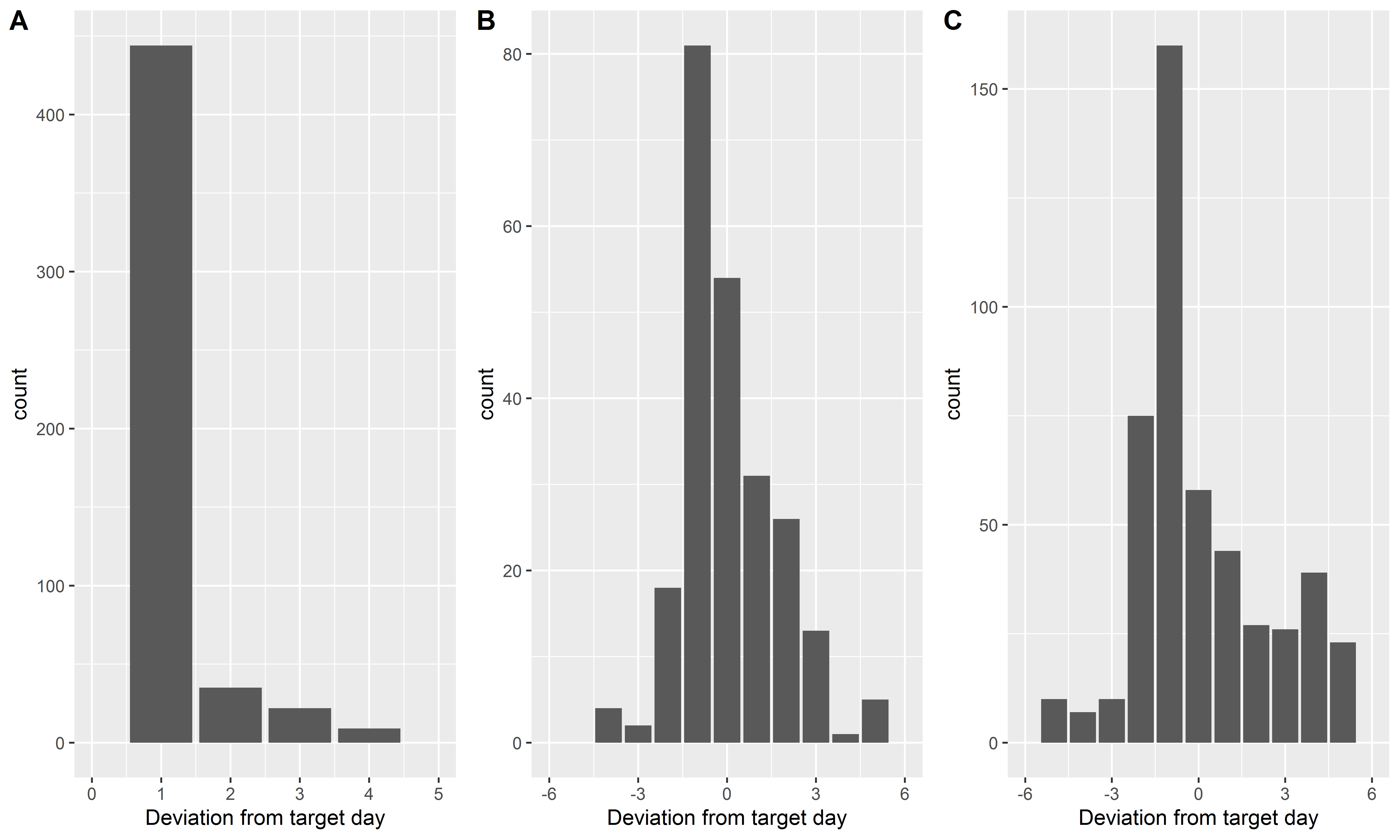


Figure S1. Plotted is the frequency distribution of deviation from the target date of physical mass measurements for pups. (A) deviation from the target parturition date, (B) deviation from the target mid-lactation date of 20 days post-parturition, and (C) deviation from target weaning date of 35 days post-parturition.


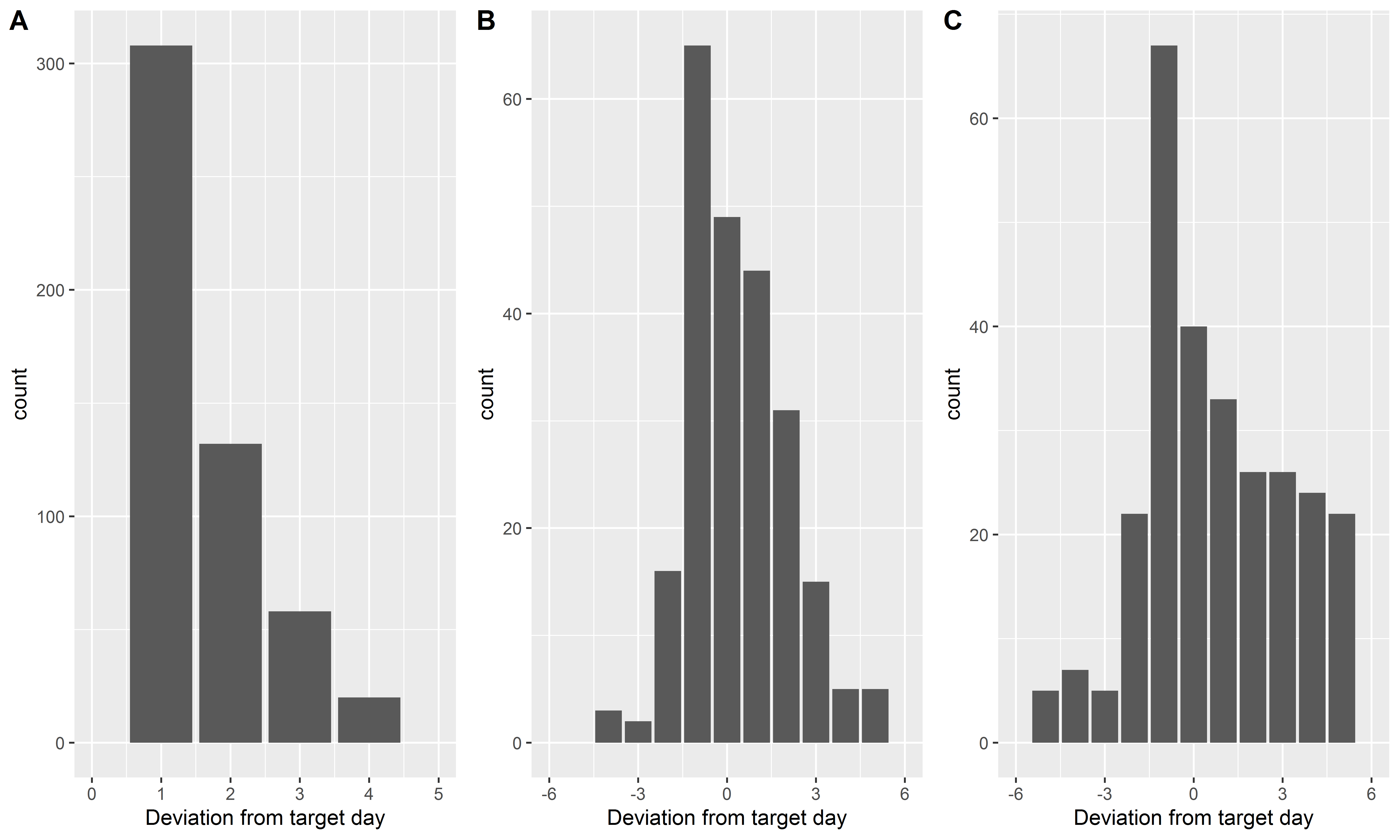


Figure S2. Plotted is the frequency distribution of deviation from the target date of physical mass and photogrammetric measurements for mothers. (A) deviation from the target parturition date, (B) deviation from the target mid-lactation date of 20 days post-parturition, and (C) deviation from target weaning date of 35 days post-parturition.

Two-dimensional photogrammetry

Photographs were collected from two perspectives, overhead and ground-level side, while the seal lay in a standard position. Two-dimensional photographs were processed using image analysis software ImageJ (Schneider, Rasband, & Eliceiri, 2012) to obtain morphometric measurements of mothers. The outline of seals on both overhead and side-view photographs were drawn excluding fore- and rear-flippers, to determine the area of the seal. Overhead width was measured as the widest distance across the chest of the seal and side-view height was measured as the longest distance from the bottom to the top edge of the seal. Due to the logistical issues associated with the two-dimensional photogrammetry technique in the field a new technique was adopted after 2010.

To identify a predictive mass-estimation equation for the two-dimensional photogrammetry, we performed all-possible-subset regression using the MuMIn package in R (Bartoń, 2018). Best supported models were chosen using the Akaike Information Criterion for small sample sizes (AIC_C_) (Burnham & Anderson, 2002). To include information from models with even moderate support, all models within five AIC_C_ units of the top model were considered as a potential predictive model (Burnham & Anderson, 2002). The subset of potential predictive models were evaluated using the predicted sum of squares (PRESS) criterion following the methodology of Ireland et al. (2006). The PRESS criterion selects for small prediction errors and identifies models with higher predictive ability (Neter, Wasserman, & Kutner, 1990). The top mass-estimation model for two-dimensional photogrammetry was selected as the model with the lowest PRESS value. Model diagnostics suggested an influential observation. Subsequent model selection was performed with and without the observation resulting in the same top model, and the observation was removed before choosing the top predictive model. The mass estimation model for the two-dimensional photogrammetry had an adjusted R^2^ value of 0.797. When the mass estimation regression was evaluated at the mean of the dependent variables (overhead area, overhead width and side-view area), the prediction error was 9.0% of the mean measured mass.

Three-dimensional photogrammetry

We took photographs from eight angles of females lying in a natural resting position and retained photograph sets for which a minimal amount of head or body movement existed among photographs in the set. Photographs were taken with Panasonic Lumix DMC-TS5 and Panasonic Lumix DMC-TS30. Photographs were processed using PhotoModeler software (Version 2016.0.8.1825, EOS Systems) to obtain three-dimensional models and volume estimates for each seal. Sets of photographs were manually oriented in three-dimensional space, silhouettes of the seal were drawn and digitally combined to obtain a three-dimensional model and subsequent volume. Morphometric measurements from photogrammetry were related to mass measurements using linear regression to obtain mass estimates.

A predictive mass-estimation equation for the three-dimensional photogrammetry was identified by performing a simple linear regression of volume versus mass. Although mass estimates from three-dimensional photogrammetry have been calculated by multiplying volume measurements and known mammal densities (de Bruyn et al. 2009, Beltran et al. 2018), such estimates typically require use of correction factors. For the mass-estimation work presented here, we performed a simple linear regression of known female masses against their respective volume estimates, which yielded a mass estimation equation specific to this population that did not require any correction factor. An influential observation was identified while assessing model diagnostics and the analysis was performed with and without the observation. Removal of the influential observation did not change the coefficient estimates of the mass-estimation model in a meaningful way, and the point was left out of the final mass-estimation model. The mass-estimation regression for three-dimensional photogrammetry resulted in an adjusted R^2^ value of 0.903. When the mass-estimation regression was evaluated at the mean volume estimate, the prediction error was 4.92% of the mean measured mass.

Appendix S2 – Models of maternal allocation

The functional forms of maternal age listed below were included in the three models of maternal allocation. All functional forms of maternal age were first assessed without including the birthdate covariate. The top model from each model suite was subsequently run including a functional form of birthdate.

Functional forms of maternal age:

| Null | $f\left( {MomAge}_{i} \right)=0$ |
| --- | --- |
| Linear | ${f({MomAge}_{i})=\beta}_{MomAge}\times{MomAge}_{i}$ |
| Logarithm | ${f({MomAge}_{i})=\beta}_{MomAge}\times{\log(MomAge}_{i})$ |
| Quadratic | ${f({MomAge}_{i})=\beta}_{MomAge}\times{MomAge}_{i}+\beta_{MomAgeQuad}\times{MomAge}_{i}^{2}$ |

Proportion allocation model:

$logit\left( \mu_{i, j,k} \right)= \alpha+f\left( {MomAge}_{i} \right)+\beta_{MomMass}\times{MomMass}_{i}+\beta_{Days}\times{Days}_{i}+\beta_{primiparity}\times{Primiparity}_{i}+\beta_{FirstTime}\times{FirstTime}_{i}+\beta_{Pre}\times{Pre}_{i}+\beta_{Skip}\times{Skip}_{i}+\beta_{Bday}\times{Bday}_{i}+\beta_{Bday.q}\times{Bday}_{i}^{2}+\beta_{Sex}\times{PupMale}_{i}+\eta_{j}+\gamma_{k}$

Mass transfer efficiency model:

$logit\left( \mu_{i, j,k} \right)= \alpha+f({MomAge}_{i})+\beta_{MomMass}\times{MomMass}_{i}+\beta_{primiparity}\times{Primiparity}_{i}+\beta_{FirstTime}\times{FirstTime}_{i}+\beta_{Pre}\times{Pre}_{i}+\beta_{Skip}\times{Skip}_{i}+\beta_{Bday}\times{Bday}_{i}+\beta_{Bday.q}\times{Bday}_{i}^{2}+\beta_{Sex}\times{PupMale}_{i}+\eta_{j}+\gamma_{k}$

Pup weaning mass model:

$\mu_{i, j,k}= \alpha+f({MomAge}_{i})+\beta_{MomMass}\times{MomMass}_{i}+\beta_{PupMass}\times{PupMass}_{i}+\beta_{primiparity}\times{Primiparity}_{i}+\beta_{FirstTime}\times{FirstTime}_{i}+\beta_{Pre}\times{Pre}_{i}+\beta_{Skip}\times{Skip}_{i}+\beta_{Bday}\times{Bday}_{i}+\beta_{Bday.q}\times{Bday}_{i}^{2}+\beta_{Sex}\times{PupMale}_{i}+\eta_{j}+\gamma_{k}$

Appendix S3 – Structure for modeling maternal mass

Three different types of mass measurements and their associated prediction errors were included in the analyses. We considered the mass of a mother that was weighed on the weigh platform to be an accurate measure of the true mass of a female and to have a prediction error of zero. For each mass estimated from two-dimensional and three-dimensional photogrammetry, we used the relevant mass-prediction regression model to obtain the mass estimate and its associated prediction error. In the subsequent Bayesian modeling of sources of variation in maternal allocation, each of our mass estimates were modeled as being normally distributed about a female’s true maternal mass and dispersed according to the associated prediction error (0.01 was added to each prediction error to avoid computational issues associated with values of 0 for prediction error).

In the proportion mass loss model, latent values of true maternal parturition mass and true maternal late-lactation mass were used to provide information on true proportion mass loss, which was then modeled as a function of covariates of interest. For the mass transfer efficiency model, latent values of true maternal parturition mass and true maternal mid-lactation mass were used to measure mass loss by mothers, pup mass measurements at parturition and mid-lactation mass provided information on daily mass gain by pups, and mass transfer efficiency (daily pup mass gain divided by latent daily maternal mass loss) was subsequently modeled as a function of covariates of interest. When modeling proportion mass loss and mass transfer efficiency, we used a logit-link between the response variable and linear functions of the covariates and regression coefficients to constrain estimated mean response values to be between zero and one. Pup weaning mass was modeled as a function of the latent value of true maternal parturition mass and other covariates of interest.

Appendix S4 – Figures of raw data and predicted relationships between maternal age and maternal allocation.


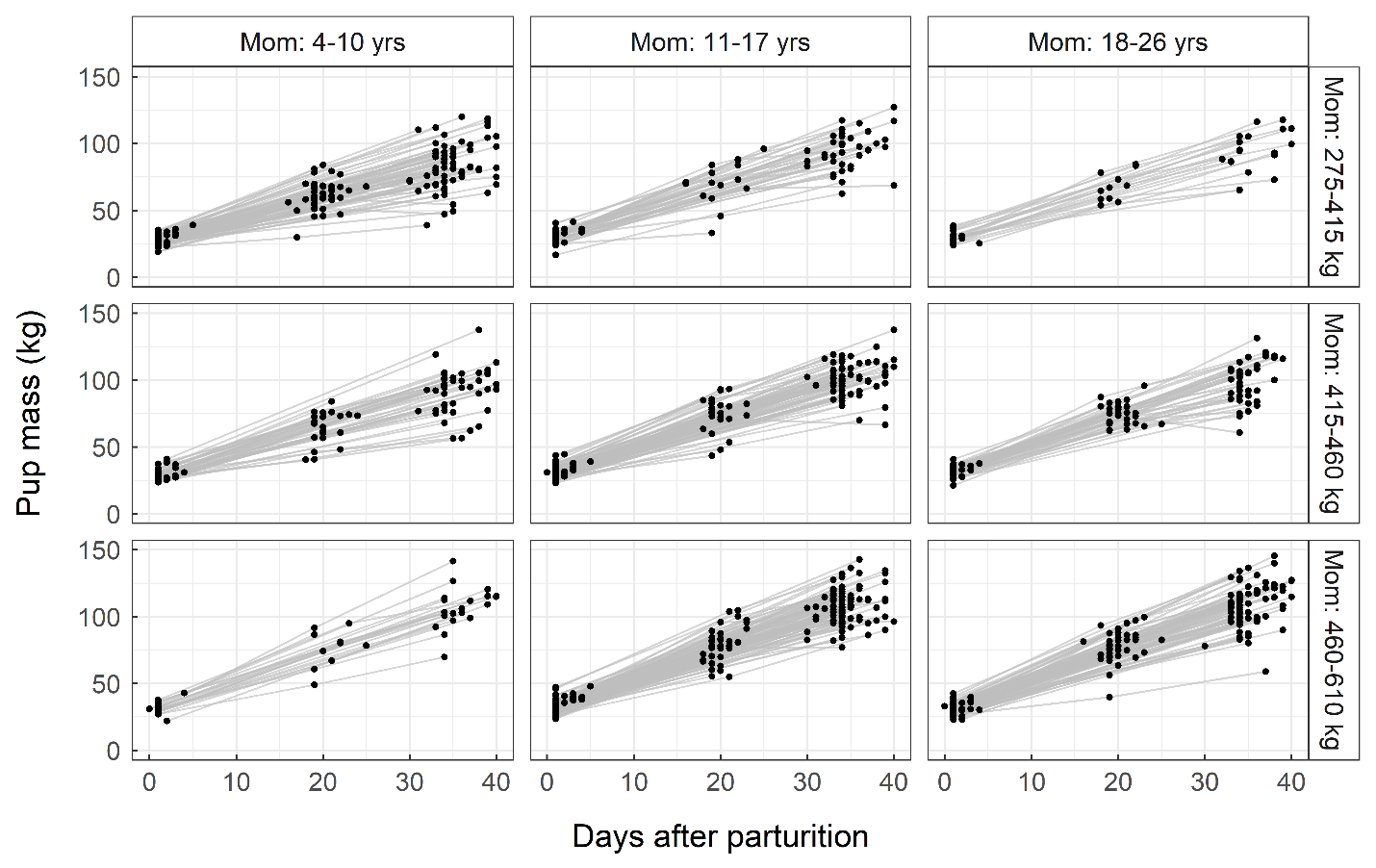


Figure S3. Masses of pups at birth, mid-lactation and weaning parturition arranged by maternal age (young, prime, old) and raw maternal mass estimates (light, average, heavy).


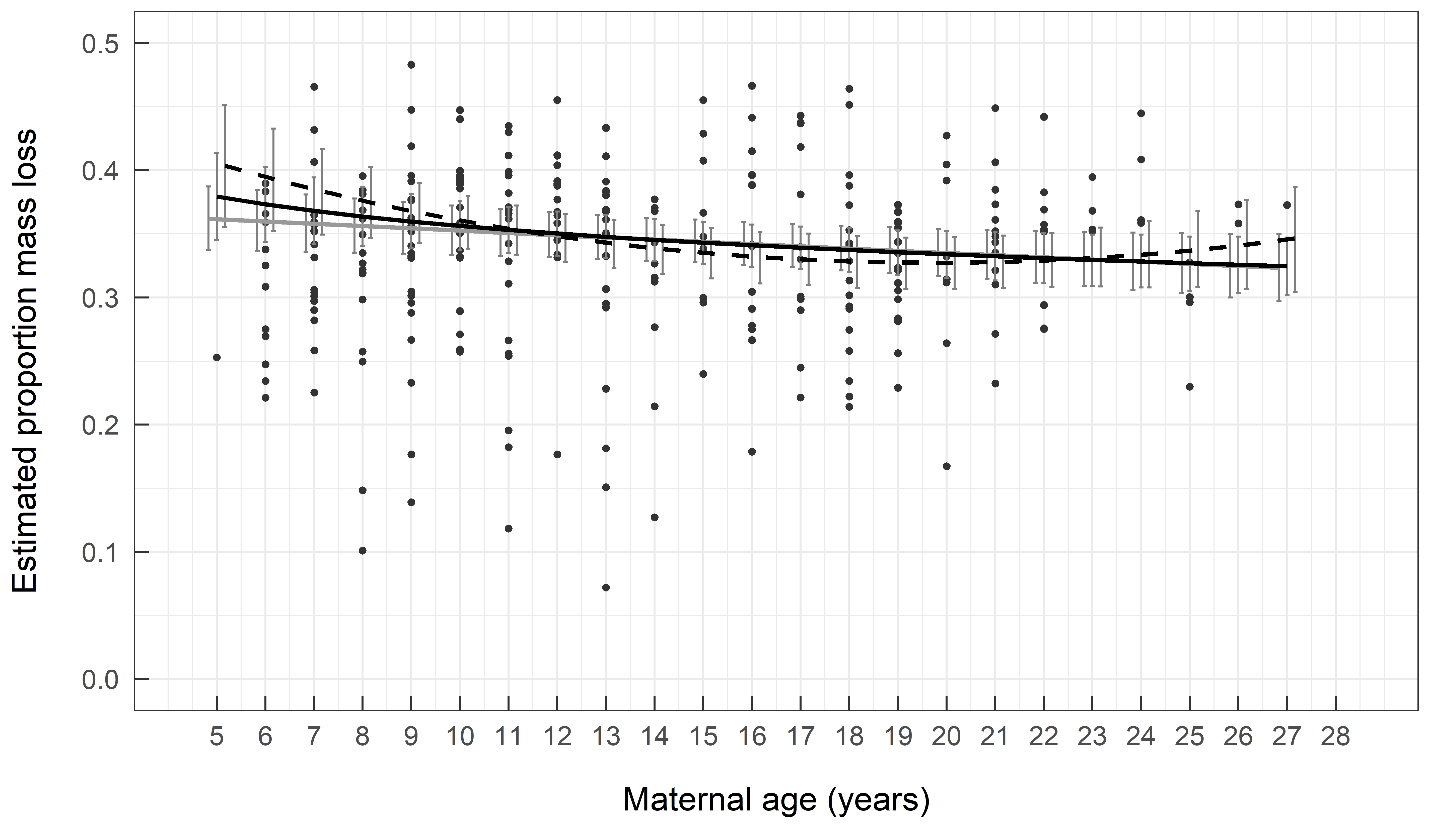


Figure S4. Comparison of the predicted relationship between proportion mass loss and maternal age for models with similar predictive ability with 90% HDIs. The log maternal age model is shown with a solid black line, the quadratic maternal age model is shown with a short-dashed line and the linear age model is shown with a grey line. The proportion mass loss of mothers calculated from the raw data before accounting for measurement error are displayed as points, the proportion mass loss of mothers was shrunk towards the mean once measurement errors were accounted for in modeling.


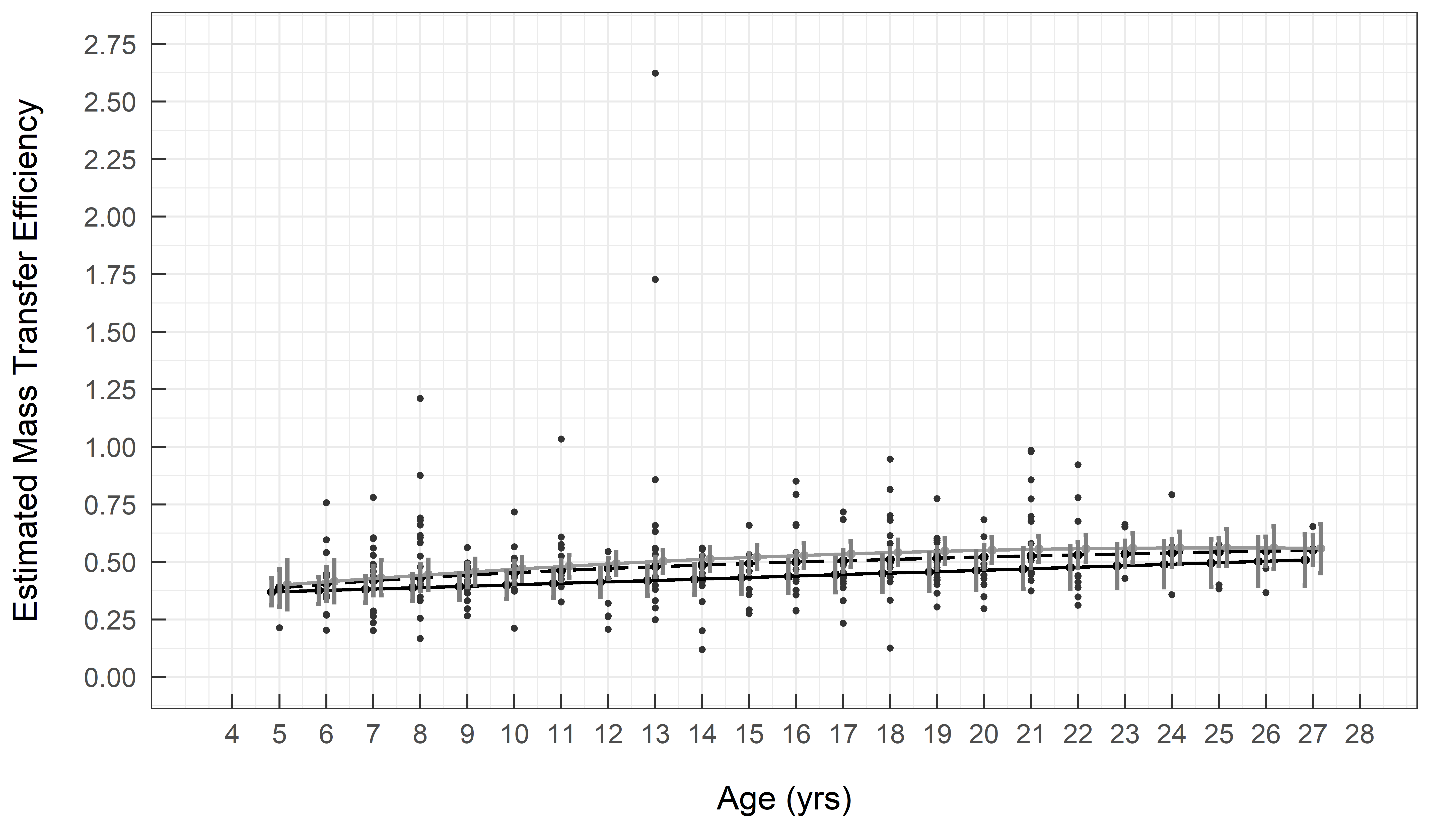


Figure S5. Comparison of the predicted relationship between apparent mass transfer efficiency and maternal age for models with similar predictive ability with 90% HDIs. The log maternal age model is shown with a short-dashed line, the quadratic maternal age model is shown with a solid grey line and the linear age model is shown with a solid black line. The apparent mass transfer efficiency of mothers calculated from the raw data before accounting for measurement error are displayed as points, the apparent mass transfer efficiency of mothers was shrunk towards the mean once measurement errors were accounted for in modeling and were estimated to be between zero and one.


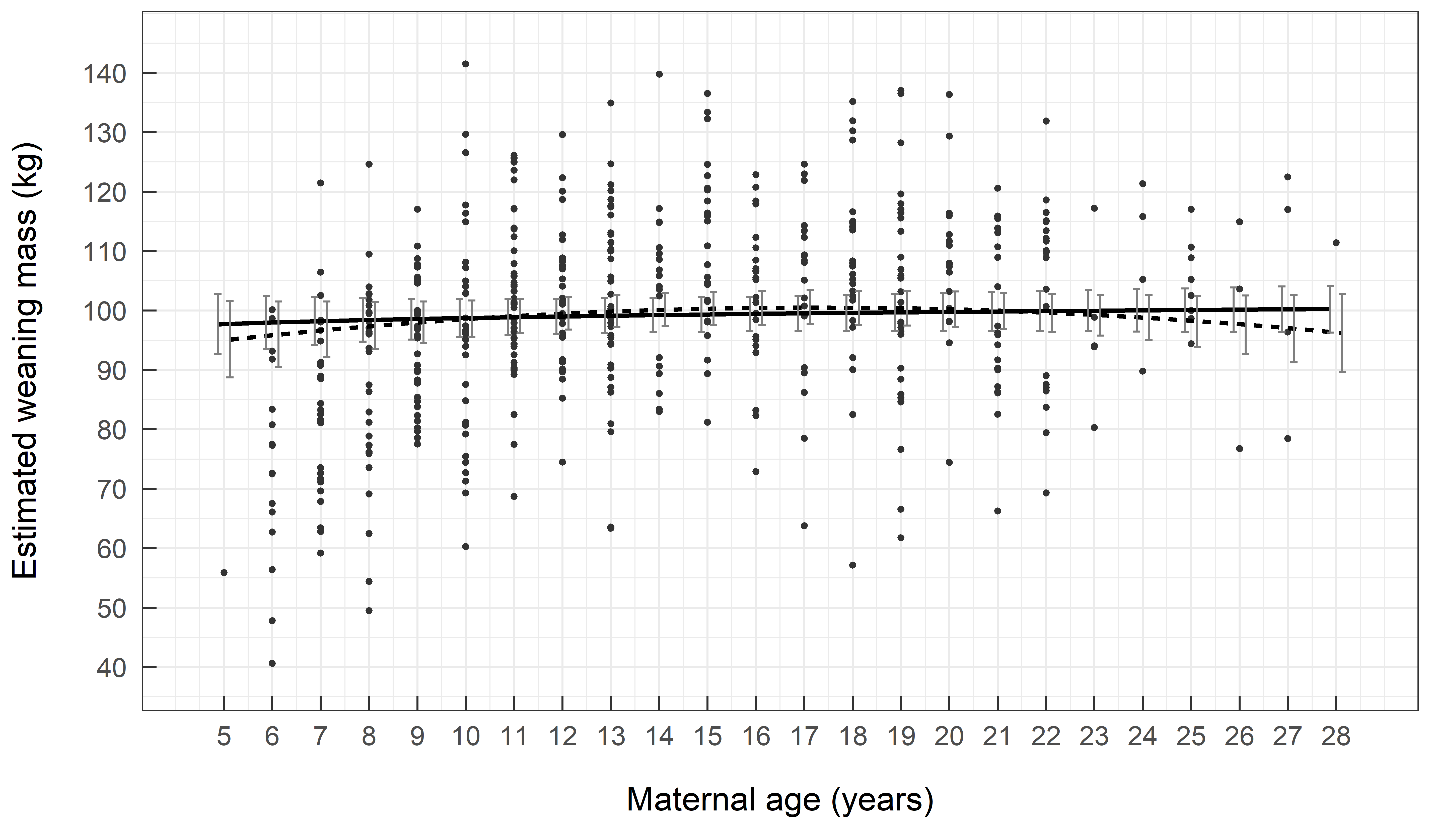


Figure S6. Comparison of the predicted relationship between pup weaning mass and maternal age for models with similar predictive ability with 90% HDIs. The log maternal age model is shown with a solid black line, the quadratic maternal age model is shown with a short-dashed line. The null model which did not contain the maternal age covariate had a similar predictive ability to the two models shown. The weaning mass of pups are plotted against their mother’s age and displayed as points.

**References**

Bartoń, K. (2018) MuMIn: Multi-Model Inference, version 1.40.4.

Beltran, R. S., Ruscher-Hill, B., Kirkham, A. L. & Burns, J. M. (2018) An evaluation of three

dimensional photogrammetric and morphometric techniques for estimating volume and mass in Weddell seals Leptonychotes weddellii. *PLOS ONE*, 13, e0189865.

Burnham, K. P., & Anderson, D. R. (2002) Model Selection and Multimodel Inference, A

Practical Information-Theoretic Approach (2^nd^ ed.). Springer, New York, New York, USA.

Ireland, D., Garrott, R.A., Rotella, J., & Banfield, J. (2006). Development and application of a mass-estimation method for Weddell seals. *Marine Mammal Science*, 22, 361-378.

Neter, J., Wasserman, W. & Kutner, M. (1990) *Applied Linear Statistical Models: Regression,*

*Analysis of Variance, and Experimental Designs* (3^rd^ ed.). Homewood, IL: Irwin

Schneider, C. A., Rasband, W. S. & Eliceiri, K. W. (2012) NIH Image to ImageJ: 25 years of

image analysis. *Nature Methods*, 9, 671-675.

de Bruyn, P. J. N., Bester, M. N., Carlini, A. R. & Oosthuizen, W. C. (2009) How to weigh an

elephant seal with one finger: a simple three-dimensional photogrammetric application. *Aquatic Biology*, 5, 31–39.
